# Reaching measures and feedback effects in auditory peripersonal space

**DOI:** 10.1038/s41598-019-45755-2

**Published:** 2019-07-01

**Authors:** Mercedes X. Hüg, Ramiro O. Vergara, Fabián C. Tommasini, Pablo E. Etchemendy, Fernando Bermejo, Laura G. Fernandez

**Affiliations:** 1 Centro de Investigación y Transferencia en Acústica (CINTRA), Universidad Tecnológica Nacional - Facultad Regional Córdoba, CONICET, 5000 Córdoba, Argentina; 2 Facultad de Psicología, Universidad Nacional de Córdoba, Argentina, 5000 Córdoba, Argentina; 3 Consejo Nacional de Investigaciones Científicas y Tecnológicas (CONICET), Buenos Aires, Argentina; 4 Laboratorio de Acústica y Percepción Sonora (LAPSo), Escuela Universitaria de Artes, Universidad Nacional de Quilmes, 1876 Bernal, Buenos Aires Argentina; 5 Present Address: Service de psychologie, Centre d’évaluation et de traitement de la douleur, Hôpital Saint-Antoine, 184, rue du Faubourg Saint-Antoine, 75012 Paris, France

**Keywords:** Perception, Human behaviour

## Abstract

We analyse the effects of exploration feedback on reaching measures of perceived auditory peripersonal space (APS) boundary and the auditory distance perception (ADP) of sound sources located within it. We conducted an experiment in which the participants had to estimate if a sound source was (or not) reachable and to estimate its distance (40 to 150 cm in 5-cm steps) by reaching to a small loudspeaker. The stimulus consisted of a train of three bursts of Gaussian broadband noise. Participants were randomly assigned to two groups: Experimental (EG) and Control (CG). There were three phases in the following order: Pretest–Test–Posttest. For all phases, the listeners performed the same task except for the EG-Test phase where the participants reach in order to touch the sound source. We applied models to characterise the participants’ responses and provide evidence that feedback significantly reduces the response bias of both the perceived boundary of the APS and the ADP of sound sources located within reach. In the CG, the repetition of the task did not affect APS and ADP accuracy, but it improved the performance consistency: the reachable uncertainty zone in APS was reduced and there was a tendency to decrease variability in ADP.

## Introduction

The peripersonal space –the immediately surrounding area in which manual exploration is performed^1,2^–constitutes a field of study which has increased interest in recent years^3^. In the peripersonal space, proprioceptive, motor and sensory information are dynamically updated to allow an accurate spatial localization of near events and objects^4–7^. Reaching, which consists in performing the action of moving the arm in order to bring the hand near a target, is a suitable ecological direct-location response to study spatial human perception performance in the peripersonal zone^8^.

While there is a large corpus of literature about reaching behaviour under visual cues, research about reaching auditory depth perception in the peripersonal space is relatively scarce^9^. That involves, on the one hand, the perception of auditory peripersonal space (APS) boundary^10^ (is a sound source within or beyond my reach?) and, on the other hand, the estimation of the sound source distance relative to the listener (how near/far is the sound source from my body?), namely auditory distance perception (ADP)^11^.

It is known that in the presence of visual information, peripersonal space boundary is slightly overestimated^12–15^ and distance perception of near visual objects is accurate^16,17^. Conversely, available data about performance in APS perception and ADP is conflictive. On the one hand, Rosenblum, Wuestefeld and Anderson^18^ used verbal report to measure whether a sound source was perceived as reachable and found a slight overestimation on performance. On the other hand, ADP literature reported a clear tendency towards overestimation of nearby sources located approximately less than 1.5 m away^19–24^. In other words, participants perceive sound sources at longer distances than the actual ones. But in terms of APS estimation, this would indicate the underestimation of its boundary, that is, participants perceive as unreachable a source that in fact, is located within reach. This type of response does not match with the accurate response reported by Rosenblum *et al*.^18^ and is contradictory to that observed in the visual modality, where participants tend to overestimate their arm’s reach.

Although both, APS boundary and ADP are functionally linked in daily reaching behaviours, they have been studied separately with different methodologies. To our knowledge, there are no previous studies that have addressed both aspects in the same experimental situation in order to make direct comparisons of the results. The first aim of this work was to measure both APS boundary and ADP in the same experimental conditions in terms of the stimulus used (by presenting a real source fixed in amplitude located on a table), the environment (by using an acoustically treated room) and the response method (by using reaching measures). Since peripersonal space operates in body-centred reference frames, the perceptual bias associated to perceive its limits and estimates near distances should be similar. Our hypothesis is that both, APS boundary and ADP, will evidence related biases.

Another aspect that has been scarcely studied is the effect of feedback in the APS and auditory distance responses. Reaching usually implies to actively touch and manipulate objects, which could serve as an appropriate way to calibrate this sensorimotor pattern. Although we have not found studies that implemented learning protocols based on reaching measures in auditory peripersonal space, there is increasing evidence that relates the plasticity of the peripersonal space boundary and the perceived visual distance with reaching as a goal-directed action.

On one hand, peripersonal space boundary extends after using a tool to reach and contact far objects^25,26^. The active use of a manual stick to visually bisect a line causes an extension of the peripersonal space, but this effect is not observed if the participant orient a laser to give their response^27^. Accordingly, subjects visually perceived the distance of objects contacted with a baton as closer than the distance of objects reached with their hand^28^. Peripersonal space boundary can also be reduced if participants use weights on their wrists when reaching a visual target, due to the effort involved in moving the arm under those conditions^29^. However, it has not been studied how the APS boundary is modulated by active feedback through reaching and touching a sound source. On the other hand, behavioural demonstrations of learning from experience in the sound localization system have been reported under both altered (i.e. by using molds in outer ears) and normal listening conditions in the horizontal plane (see^30,31^ for a review). The study of audio tactile interaction has revealed the existence of a spatial modulation between both modalities and has showed that passive tactile information can modify the perception of a sound source position in the horizontal plane (see review in^32^). Even though it has been mentioned that active tactile exploration could be used to calibrate auditory perception of distance^9^, as far as we know this aspect has not been previously studied in the peripersonal space.

The second aim of our study was to measure the effect of feedback on both APS boundary and ADP responses. Because both tasks are functionally related, we hypothesise that feedback will improve performance in both of them. Taking into account previous visual studies on APS boundary, we suppose that allowing participants to explore the environment to contact the sound source will adjust its limit to their maximum reachable distance. We expected also that ADP bias will be reduced because active exploration will provide feedback about the actual position of the sound source.

In summary, the aim of the present study was twofold: to measure the APS boundary and near field ADP with a reaching response without visual cues, and to analyse the effects of feedback on performance. A randomized control-group pretest-posttest design was implemented and participants resolved both tasks under similar conditions and in a single experiment.

## Methods

### Testing environment

The experiment was performed in an acoustically treated room (4.20-m long, 3.80-m wide and 2.60-m high; volume ~41 m^3^) with walls and ceiling covered by fiberglass panels and the floor by carpet. The reverberation time was 170 ms at 1 kHz octave band. The background noise was ~17 dBA SPL.

### Participants

Twenty adults participated in the experiment (10 women, mean age = 26.6, SD = 3.7). All participants were right-handed and reported no hearing problems. None had previous knowledge of the set-up nor were informed about any characteristic of the room. The study was carried out in accordance with the Helsinki Declaration guidelines. The protocol was evaluated and approved by the Institutional Ethics Committee (CIEIS Hospital Nacional de Clínicas, Universidad Nacional de Córdoba). All participants provided written informed consent prior to the beginning of the experiment.

### Experimental set-up

**Figure 1:**
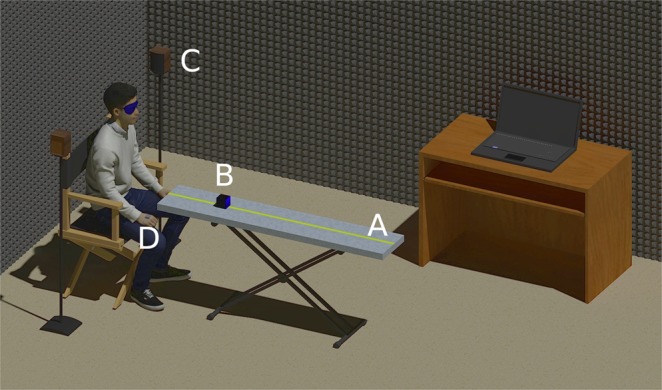
Illustration of the experimental set-up: table placed on a support of adjustable height (**A**), sound source (**B**), masking sound system (**C**), motion tracker mounted on the back of the participant’s hand (**D**).

The experimental set-up consisted of a wooden table (150-cm long, 30-cm wide) placed on a metal support of adjustable height (Fig. 1A). The table was covered with a sound-absorbing material and lined with fabric. The height of the experimental table was regulated 40 cm below the participant’s ears. Twenty-three target distances were used (40 to 150 cm in 5-cm steps, measured from the participant’s body vertical axis). The vertical angles from the subject’s head at the nearest and farthest distances were −45° and −14.9°, respectively (see Supplementary Table S1). The experimenter (not shown in Fig. 1 for clarity) was located next to the table, on the right side of the participant and behind the sound source. He/she moved manually the speaker (Sony SRS-X11; 6.1 × 6.1 × 6.1 cm; Fig. 1B) over the table located in front of the participant (0° azimuth) according to the distance indicated by a software on the computer screen. A purpose-built rule containing all the test distances was fixed to the table.

The sound stimulus was based on Macé, Dramas and Jouffrais^33^ study. It consisted of a train of three bursts of Gaussian broadband noise (0.02–22 kHz), 100-ms-long each one, with onset and offset ramped by 2-ms half-Hamming windows and 50 ms of silence between bursts. The stimulus total length was 400 ms.

Stimulus sound level was calibrated to a comfortable level of approximately 65 dBA SPL measured at the participant’s head position with the sound source located at 1 m (see Supplementary Table S1 for details). The availability of distance cues in the environment was not constrained. The level of the sound source was fixed, so as to allow sound intensity at the listener’s ears to vary naturally with source distance.

Between trials, a masking sound was presented through two loudspeakers located at both sides of the participant (Fig. 1C) at a similar sound level to that of the stimulus. One second after the end of the masking sound, the auditory stimulus was presented through the test speaker. Speakers were controlled by an audio interface (Creative E-MU 0404).

Participant’s hand position was captured with a motion tracker (Polhemus Patriot) mounted on the back of the hand on the knuckle of the middle finger (Fig. 1D). A specific software was developed using MATLAB (MathWorks) that allowed to manage the experiment and the data collection.

### General procedure

Each participant started the experiment in the reception room where he/she received initial instructions on the task. Afterwards, the experimenter measured the size of his/her dominant hand. Then, the participant was blindfolded and led into the test room.

A randomized control-group pretest-posttest design was implemented. Participants were randomly assigned to two groups: Experimental (EG) (10 subjects, 5 women) and Control (CG) (10 subjects, 5 women). Each participant performed three phases in the following order: Pretest–Test–Posttest. The sound source was presented at one of 23 target distances in each trial. Participants were asked to extend their arm and put their hand on the table in the place where they perceived the source. If necessary, they could lean his/her body forward without raising from the chair (based on^12,18^). If they considered that the source was beyond reach, they should respond by saying “no”. Participants were asked to respond with his/her dominant hand.

In the Pretest and Posttest phases, the experimenter gently removed the source from the table immediately after the stimulus ceased, and subjects did not receive any feedback about their performance. In the Test phase, the sound source was not removed for the EG, and participants were encouraged to explore their frontal space until the source was touched, obtaining in this way feedback about its position. To ensure correct feedback in this phase, if a participant said “no” but the source was reachable, the experimenter asked the subject to explore the table until to find the source. Conversely, the CG repeated the same task performed in Pretest and Posttest without any feedback.

Before starting the experiment, the maximum reachable distance (MRD) of each participant was obtained by asking the subject to lean forward as much as possible without raising from the chair, and to extend his/her arm and hand on the table. MRD was measured at the end of the middle finger position. Participants completed initially a familiarisation session of 4 trials executed at reachable and unreachable distances within the 23 possible ones, in which no feedback was provided. Two trials were randomly selected from distances between 40 cm and the MRD of the participant, and the 2 other trials were randomly chosen from distances between his/her MRD and 150 cm.

In the experiment, each target was presented 4 times in random order. All distances were presented one time before the next repetition, giving a total of 92 trials (4 repetitions × 23 distances). Each phase lasted about 20 min. To avoid fatigue, both groups took a short break of 5 min between Pretest and Test phases.

### Data analysis

#### Perceived reachability boundary: within-beyond reaching attempts

The targets were classified into two categories: *within* and *beyond* reach, using the MRD as criterion. As the MRD differed across subjects, this classification was done separately for each participant. The number of targets in each category was balanced: as our experimental setup has the majority of targets within reachable distance, the number of *beyond*-reach target positions was taken as a reference and the same number of *within*-reach positions closer to the MRD was included for the analysis, discarding the remaining ones. The proportion of reach attempts *p*_*ijk*_ was calculated as the total number of reach attempts performed by participant *i* in phase *j* (*j* = Pretest, Posttest) in response to targets pertaining to category *k* (*k* = *within*-reach, *beyond*-reach) divided by the total number of target presentations. For the purpose of averaging and statistical modelling of the data, the Fisher transformation was applied, as it improves normality: $z={\rm{atanh}}(2p-1)$, where *p* is the proportion of reach attempts and *z* the transformed variable. Statistical analysis was carried out employing a repeated-measures ANOVA on the transformed proportions (see Results for a description of the ANOVA).

#### Perceived reachability boundary: logistic model

Previous studies on reachability in the peripersonal space showed that arm length affect participants’ performance^12,18,34^. For this reason, responses were normalized according to every MRD, i.e. the ratio of the actual distance to MRD was calculated for each participant, leading to a normalized distance scale. A logistic regression model was proposed to characterise perceived reachability boundary of APS. This model follows an inverted sigmoidal path that captures the expected gradual transition from initial reaching attempts values for a sound source very close to the participant’s body to the lack of reaching attempts at far distances. Normalized distance intervals (bins) were created and the mean of reaching attempts within each interval was calculated^35^. The width of the interval was determined by estimating the normalized distance proportion in which each participant contributed with at most one (not normalized) distance position per bin (see Supplementary Table S2).

In line with previous studies of visual perception of peripersonal space^36,37^, the proportion of reaching attempts as a function of normalized reaching distance was fitted to the logistic function, which is defined as a special form of the Richards’ function

1 $${Y}_{x}=\frac{A}{1+{e}^{-B(x-M)}}$$

where *Y*_*x*_ is the reaching attempts ratio, *x* is the normalized distance, and *A*, *B* and *M* are unknown estimated parameters. Fitted parameters values were calculated using initial values *B* = 0 and *M* = 1 (with *A* = 1 as fixed parameter) and a weighted function ${w}_{i}=1/{\sigma }_{i}$ for each bin, where ${\sigma }_{i}$ is the standard deviation for the *i*-th bin. According to this model, *A* is the maximum reaching attempts proportion, *B* is a rate parameter that defines the steepness of the curve, and *M* is the normalized distance of the mirrored sigmoid’s midpoint (inflection point). In this study, *M* represented the perceived MRD, measured in normalized distance units. In this point, participant responded at chance level. Other parameter is the Reachable Uncertainty Zone that was defined as the distance in which the percentage of reaching attempts were between 75% (beginning of uncertainty zone, ${x}_{Y(.75)}$) and 25% (ending of uncertainty zone, ${x}_{Y(.25)}$). The width of this zone ${\rm{\Delta }}z$ was calculated as follows: ${\rm{\Delta }}z={x}_{Y(.25)}-{x}_{Y(.75)}$, and was measured in normalized distance units. It is important to note that *M* is the midpoint of the uncertainty zone.

#### Auditory distance estimation: perceived distance

The estimated perceived distance was determined using the position provided by the motion tracker at the moment in which the participant ceased the movement of his/her hand and kept it still on the table, after making the adjustments he/she considered necessary. In this case the sensor speed value was ~0 cm/s.

Due to the observation that, in the Test phase for the EG (with feedback) subjects tended to reach the source with their fingertips when it was located up to two positions closer from his/her MRD, while they reached it with the palm for the remaining distances, a correction of the motion tracker values was implemented for such extreme distances. In those distances, the position values were corrected by adding the length from the sensor to the fingertips.

The trials in which the sound source was perceived as unreachable were coded assuming that the participant perceived the target at the first distance beyond his/her MRD. For this reason, data for targets with less than two reaching attempts were discarded from the analysis. In this way, a balance between data reliability and maximization of the target response range was obtained. Supplementary Fig. S1 illustrates the application of the criteria for each participant.

Finally, a comment is in order on the ADP data collection method. When the loudspeaker was perceived as not reachable, the participant did not perform any reaching action, and therefore ADP responses were not collected. The rationale for this design was twofold. Firstly, one of the aims of our study was to measure the effect of feedback on both ADP and APS responses. As feedback was provided by reaching with the hand, sources beyond reaching distance provide less information than sources within: the participant can learn that a far source is beyond reach, but cannot ascertain its real position because it is unreachable. For this reason, we focused on the target range for which the subject could obtain the same feedback for all tested distances. Secondly, although we could measure ADP for sources beyond reach employing alternative methods, there is evidence that visual perception distance is affected by the use of tools that artificially extend the arm length^28^.

#### Auditory distance estimation: response variability

Intra-subject variability was analysed by obtaining the standard deviation of individual responses for each target distance. Standard deviations were obtained from the mean variance across phases (i.e., the RMS of the standard deviation).

#### Auditory distance estimation: response bias

The response bias was analysed by computing the Signed Percentage Error, defined as ${\rm{SPE}}=(Y/X-1)\,\times 100 \%$, where *X* and *Y* are the actual and perceived distance, respectively. The signed percentage error for each participant in both Pretest and Posttest was collapsed across all source distances. The Unsigned Percentage Error, defined as the absolute value of the signed percentage error (see^38^), was also calculated, and it was also collapsed across all distances.

## Results

### Perceived reachability boundary

#### Within-beyond reaching attempts

The first analysis aims to characterise the effect of exploration on the perceived reachability for sound sources normalized to participant´s MRD (range of variation 110–125 cm, mean 117 cm). Average proportion of attempts were high on positions *within* the participant’s MRD (*within*-reach) for both groups (EG-Pretest: 0.92, SD = 0.11; EG-Posttest: 0.85, SD = 0.12; CG-Pretest: 0.91, SD = 0.10; CG-Posttest: 0.98, SD = 0.03) and decreased on positions *beyond*-reach (EG-Pretest: 0.58, SD = 0.24; EG-Posttest: 0.32, SD = 0.18; CG-Pretest: 0.52, SD = 0.27; CG-Posttest: 0.57, SD = 0.24). A mixed ANOVA was performed with Reachability (*within*-reach, *beyond*-reach) and Phase (Pretest, Posttest) as the within-subjects factors, and Group (EG, CG) as the between-subjects factor. Results evidenced a significant effect for Reachability [*F*(1, 18) = 220, *p* < 0.0001], indicative of a greater tendency, for all participants, to reach for the sound source when it was within MRD. A significant effect was also found for the Group × Phase interaction [*F*(1, 18) = 6.2, *p* = 0.023] showing that exploration feedback affected participant’s performance: reach attempts of EG were reduced in Posttest. The Reachability × Group interaction [*F*(1, 18) = 7.97, *p* = 0.011] evidenced that the amount of reach attempts for *beyond*-reach distances decreased more in the EG than in the CG, while responses for *within*-reach distances did not change.

#### Logistic model

In order to characterise the participant’s reachability response in the boundary of their APS, a logistic model was applied (see Methods for more details). The logistic model predicted values for Pretest phase with all participants (*N* = 20) were compared to the experimental data. The *B* parameter (steepness of the curve) was −7.8 (95% CI [−8.5, −7.2]) and *M* parameter (perceived MRD) was 1.175 MRD (95% CI [1.165, 1.185]), i.e. 17.5% beyond the actual MRD. Both estimated parameters (*B* and *M*) were not correlated (*r* = 0.170). The goodness of fit for the function was statistically compared. The residual sum of squares (RSS) was 0.0192 while the total sum of squares (TSS) was 1.92. Also, the coefficient of determination *R*² = 0.990 and the root-mean-square error RMSE = 0.0147. In summary, the model provides a good fit to experimental data (Supplementary Fig. S2).

**Figure 2:**
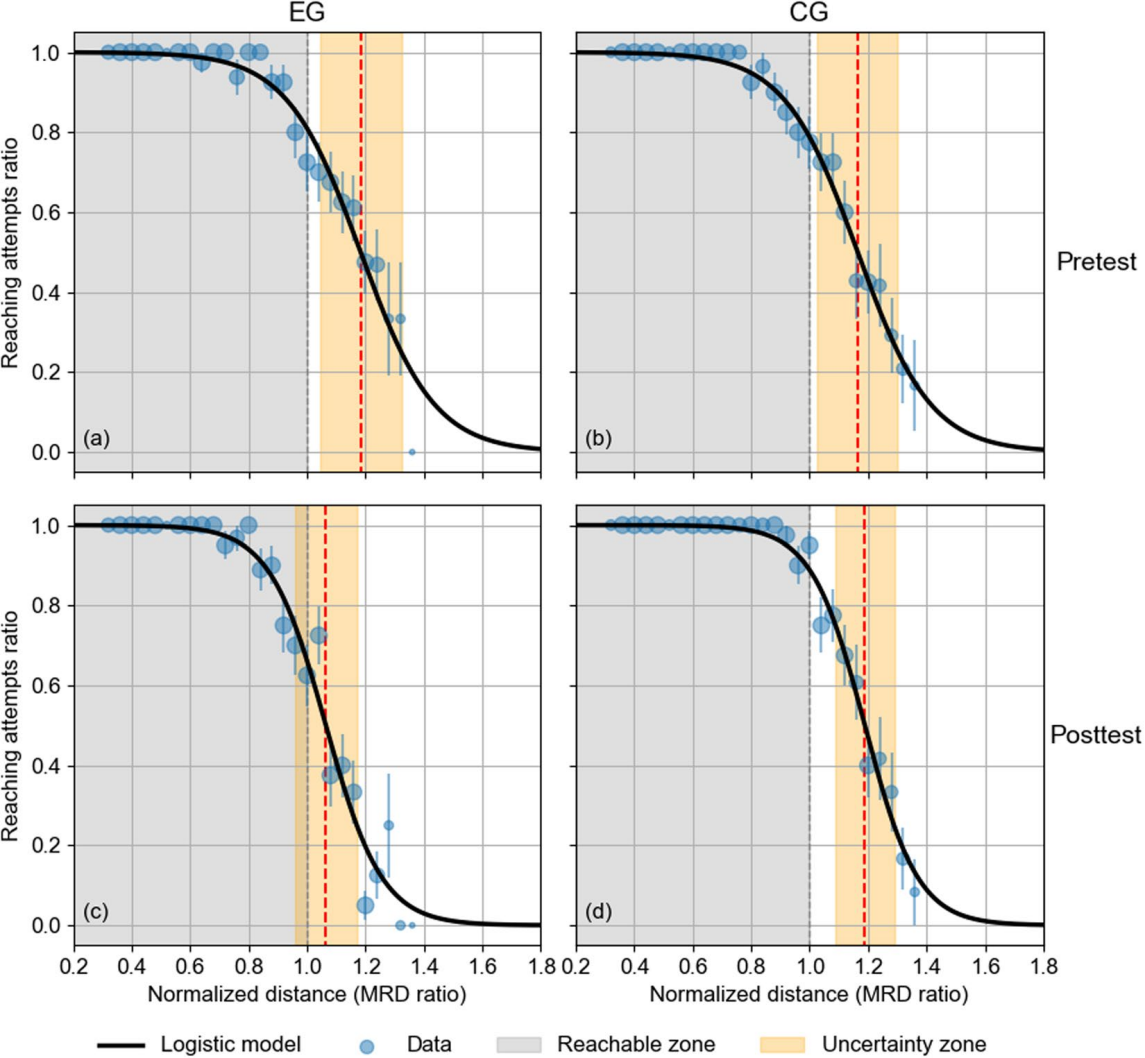
Logistic model (dark black solid line) fitted to the average of reaching attempts ratio of normalized reaching distance (MRD ratio) for (**a**) EG-Pretest, (**b**) CG-Pretest, (**c**) EG-Posttest, and (**d**) CG-Posttest. Data are presented as bubble scatter plots with SEM errors bars. Grey dashed line shows the MRD (equal to 1 in normalized distance units) and the grey area is the reachable zone. Orange zone depicts the uncertainty zone and red dashed line the perceived MRD (reaching distance at the curve inflection point).

The logistic regression was applied to the average responses for all participants in each Group and Phase (Fig. 2). The model showed a good fit to the data (*R* range 0.98–0.99). For EG, in the Pretest, *B* was −7.9 (95% CI [−9.2, −6.6]) and *M* was 1.18 MRD (95% CI [1.163, 1.204]), while in the Posttest, *B* was −10 (95% CI [−12.4, −8.5]) and *M* was 1.06 MRD (95% CI [1.045, 1.084]). On the other hand, CG showed the following results. In the Pretest, *B* was −8.0 (95% CI [−8.6, −7.3]) and *M* was 1.17 MRD (95% CI [1.154, 1.175]), while in the Posttest, *B* was -11 (95% CI [−12.2, −9.8]) and *M* was 1.19 MRD (95% CI [1.179, 1.199]).

Reaching attempt responses of each participant for each Group and Phase were fitted to the logistic function for inferential analysis function (*R* were in range 0.76–0.99, except for two participants in Pretest, for which *R* = 0.65). The initial parameters values were the previously indicated ones (see Methods section) and the weighted function was not applied. A statistical outlier’s analysis was implemented, and three participants were excluded from the sample because their responses exceeded 99% confidence interval for parameters values (see Supplementary Fig. S3), leaving a total of 17 participants (9 for the EG and 8 for CG).

Table 1 shows the mean values, (standard deviation) and [95% confidence interval] of the parameters *B* (steepness of the curve), *M* (perceived MRD) and *∆z* (width of the reachable uncertainty zone) derived from the individual fits of logistic model for each Group and Phase.

**Table 1:** Mean values, (standard deviation) and [95% confidence interval] of the parameters *B* (steepness of the curve), *M* (perceived MRD) and *∆z* (width of the reachable uncertainty zone) derived from the individual fits of logistic model for each group and phase.

Group	Perceived MRD (*M*)		Steepness of the curve (*B*)		Width of the reachable uncertainty zone (∆*z*)	
	Pretest	Posttest	Pretest	Posttest	Pretest	Posttest
EG	1.17 (0.12) [1.08, 1.26]	1.06 (0.10) [0.98, 1.14]	−9.48 (3.27) [−11.99, −6.97]	−14.62 (4,9) [−18.38, −10.85]	0.26 (0.09) [0.18, 0.33]	0.16 (0.05) [0.12, 0.21]
CG	1.16 (0.16) [1.02, 1.29]	1.17 (0.08) [1.10, 1.25]	−14.18 (4.99) [−18.35, −10.0]	−14.26 (3.62) [−17.29, −11.23]	0.17 (0.06) [0.12, 0.22]	0.16 (0.04) [0.13, 0.20]

The perceived MRD (*M* parameter) for both groups had similar initial values, and decreased for Posttest only in the case of EG (EG: *M* correction = −0.11 or −9.4%; CG: *M* correction = 0.01 or + 0.9%). These results show that the APS boundary is shifted towards the participant after exploration feedback is performed. Besides, steepness of the curve (*B* parameter) showed different Pretest mean values between groups, which became similar in the Posttest (and similar to CG-Pretest) (EG: *B* correction = −5.4 or + 54%, CG: *B* correction = −0.08 or + 0.6%). The mean values of width of the reachable uncertainty zone (*∆z*) also had a similar behaviour: were different between groups in Pretest and became similar in Posttest (EG: *∆z* correction = −0.1 or −39%, CG: *∆z* correction = −0.01 or −5.9%).

A mixed ANOVA was performed on the parameters *B*, *M* and ∆*z* values (see Data Analysis for description); with Group (EG, CG) as the between-subjects factor and Phase (Pretest, Posttest) as the within-subjects factor. No Phase effects were found in case of *B* [*F*(1, 15) = 2.619, *p* = 0.126] and *M* [*F*(1, 15) = 2.856, *p* = 0.112]; nor for interaction Phase × Group in case of *B* [*F*(1, 15) = 2.446, *p* = 0.139] and ∆*z* [*F*(1, 15) = 3.187, *p* = 0.094]. Significant effects were found on *M* for the interaction Phase × Group [*F*(1, 15) = 5.545, *p* = 0.033]. Phase affected *M* values on the Posttest only for the EG [*F*(1, 8) = 8.496, *p* = 0.019] but not for the CG [*F*(1, 7) = 0.214, *p* = 0.657]. Group, otherwise, affected *M* values only in Posttest [*F*(1, 15) = 5.774, *p* = 0.030] but not in Pretest [*F*(1, 15) = 0.043, *p* = 0.838]. Regarding to ∆*z*, significant main effects were found for Phase [*F*(1, 15) = 4.985, *p* = 0.041]. In summary, these results indicated that changes on perceived MRD (*M* parameter) were not due to the repetition of the task but to the exploration feedback performed in EG. In contrast, for the uncertainty zone (∆*z*), Posttest values were lower than the Pretest values regardless of the Group, indicating that the repetition of the task could reduce it.

### Auditory distance estimation

#### Perceived distance

**Figure 3:**
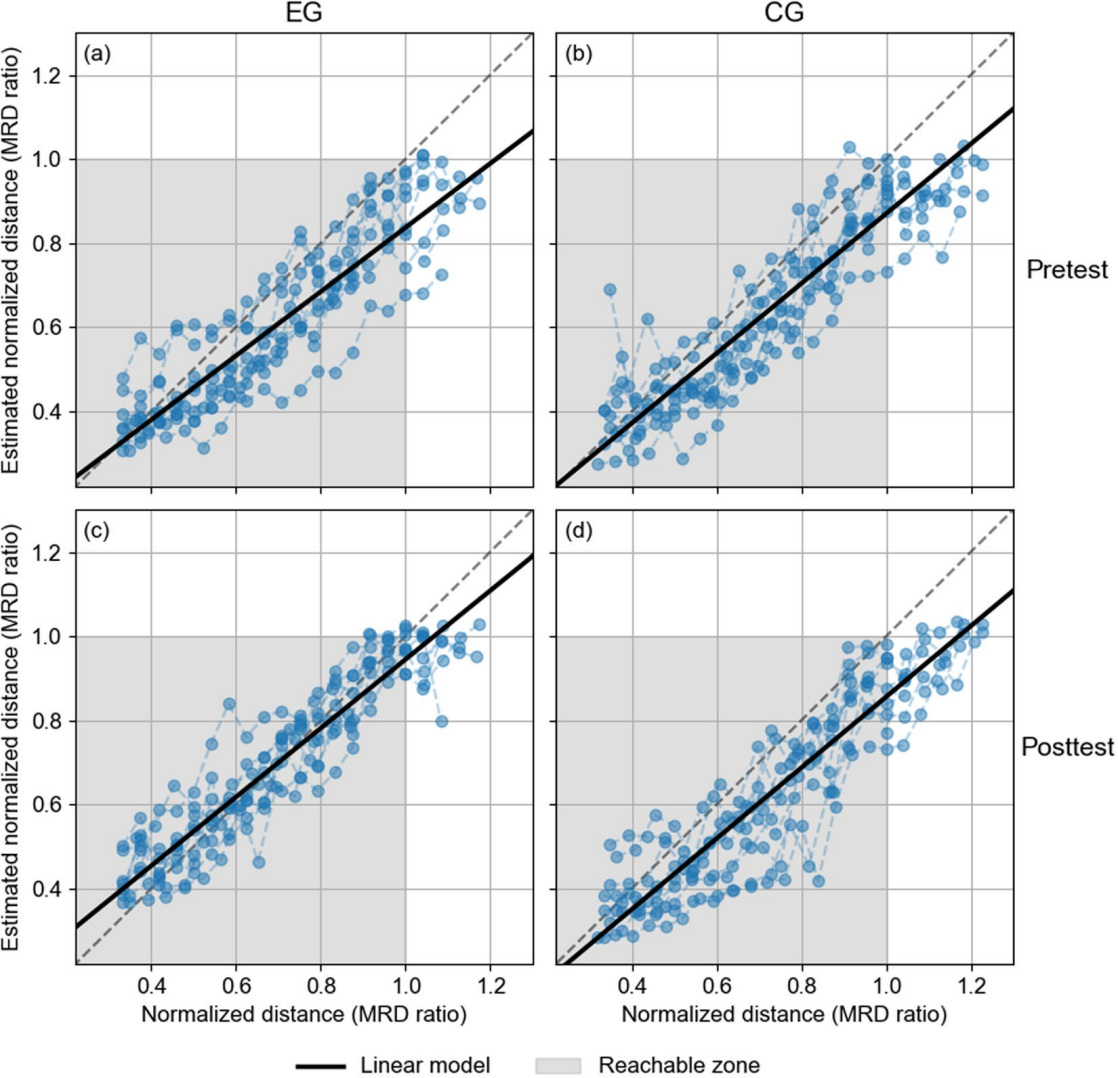
Linear model (dark black solid line) fitted to the estimated normalized distance (MRD ratio) for (**a**) EG-Pretest, (**b**) CG-Pretest, (**c**) EG- Posttest, and (**d**) CG-Posttest. Data are presented as scatter plots. Grey dashed line shows the ideal response and the grey area is the reachable zone.

Figure 3 shows the distance estimates for both groups in Pretest and Posttest, measured relative to the MRD. When subjects performed the task without previous exploration feedback (CG in both Pretest and Posttest, EG in Pretest), they showed a similar pattern of response: on average, participants perceived accurately the distances of targets nearer than ~0.4 MRD, while targets located farther from this distance were systematically underestimated.

On the other hand, after receiving feedback (EG in Posttest), subjects overestimated the perceived locations of nearest targets (up to ~0.5 MRD). The region of systematic underestimation shifted towards the region beyond ~0.9 MRD. Furthermore, targets between ~0.5 to ~0.9 MRD were perceived more accurately than in the Pretest phase, in which they were mostly underestimated by all subjects.

In order to measure the effect of feedback, individual linear fits on the average distance estimates as a function of source distance were performed (*R* range 0.90–0.99, except for one participant in Pretest, for which *R* = 0.75). Table 2 shows the mean values, (standard deviation) and [95% confidence interval] of the slope and the intercept derived from the individual fits of linear model for each group and phase.

**Table 2:** Mean values, (standard deviation) and [95% confidence interval] of the slope and the intercept derived from the individual fits derived from the linear individual fits for each group and phase.

Group	Slope		Intercept	
	Pretest	Posttest	Pretest	Posttest
EG	0.762 (0.136) [0.678, 0.847]	0.818 (0.111) [0.750, 0.887]	0.075 (0.092) [0.018, 0.132]	0.127 (0.087) [0.073, 0.181]
CG	0.830 (0.191) [0.711, 0.948]	0.842 (0.140) [0.755, 0.929]	0.040 (0.134) [−0.043, 0.123]	0.014 (0.114) [−0.057, 0.084]

The changes between Pretest and Posttest for EG evidenced that subjects shifted the responses upward (intercept correction = 0.052 MRD or +69%) practically without changes in the response range (slope correction = 0.056 or +7.3%). Although insufficient for achieving perfect performance for all targets, this correction was good enough to allow very accurate responses for most targets in the range [~0.5, ~0.9] MRD. The changes from CG in both parameters across phases was 0.012 for the slope (+1.4%) and −0.026 MRD (−65%) for the intercept.

The distance responses were analysed by means of a linear mixed-effects model on the normalized response, with fixed factors Target Distance (within-subjects), Phase (Pretest, Posttest; as within-subjects) and Group (EG, CG; as between-subjects) and random factors given by individual random intercepts and slopes. The analysis showed a significant effect of main factors Target Distance [*F*(1, 706) = 765, *p* < 0.0001] and Phase [*F*(1, 706) = 59.8, *p* < 0.0001] and of the interaction term Group × Phase [*F*(1, 706) = 136, *p* < 0.0001]. The significant effect of Group × Phase is consistent with the previous observation that subjects from EG shifted the intercept while maintaining the slope of the relation between source location and perceived distance.

Another interesting aspect is given by the inference of the perceived MRD based on the distance model. When calculating the intersection of the linear model with the value 1 of the estimated normalized distance, the perceived MRD was: EG-Pretest = 1.21, EG-Posttest = 1.07, CG-Pretest = 1.16, CG-Posttest = 1.17. These values were similar to those obtained through the analysis of the logistic curve (see Table 1).

#### Response variability

Analysis of the standard deviation showed no clear sign of improvement in the consistency of the response of EG subjects after receiving feedback (see Supplementary Fig. S4 and Supplementary Fig. S5). The responses of subjects from CG had a large variability during Pretest, which was reduced during Posttest to values in the range of that displayed by the EG. Analysis by means of a linear mixed-effects model with fixed factors Group and Phase, and random ordinates by participant, confirmed these observations: the Group × Phase interaction was significant [*F*(1,18) = 5.69, *p* = 0.028], while main factors Group and Phase were not [Group: *F*(1,18) = 0.446, *p* = 0.51; Phase: *F*(1,18) = 1.52, *p* = 0.23].

#### Response bias

**Figure 4:**
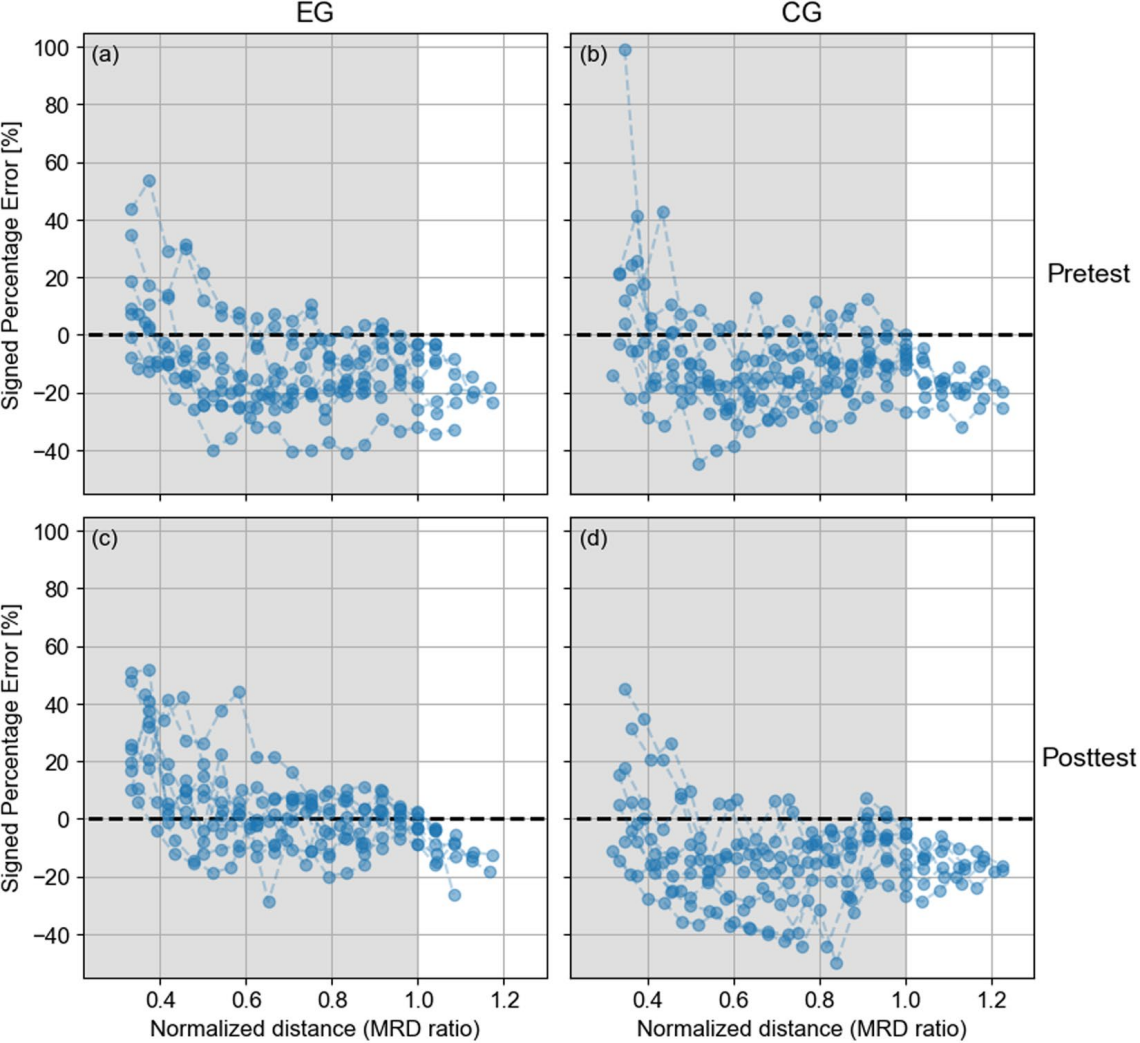
Individual signed percentage error as a function of normalized source distance (MRD ratio) for (**a**) EG-Pretest, (**b**) CG-Pretest, (**c**) EG Posttest, and (**d**) CG-Posttest. Grey area is the reachable zone.

The individual signed percentage error was plotted in Fig. 4 as a function of normalized source distance. In the Pretest, subjects from EG displayed differences in bias, between on average ~15% overestimation for the first target and 15–20% underestimation for targets beyond ~0.55 MRD, which remained almost constant for farther positions. In the Posttest, the bias changed in two aspects. Firstly, the responses for targets closer than ~0.5 MRD were more overestimated than in the Pretest (~15% in Pretest vs ~25% in Posttest). Secondly, the bias for targets between ~0.5 and ~0.9 MRD was mostly concentrated between ±10%, indicating that the subjects (considered as a whole) improved ADP accuracy in that region with respect to Pretest. Targets farther than ~0.95 MRD displayed a slight underestimation. Finally, subjects from CG displayed biases very similar to EG-Pretest’s data, independently of the phase considered.

**Figure 5:**
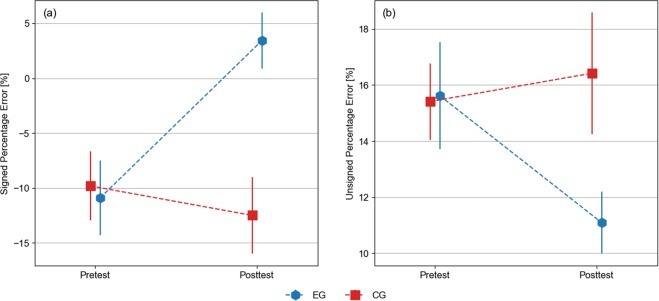
(**a**) Mean signed percentage error. (**b**) Mean unsigned percentage error. In both panels the errors correspond to the between-subject average (±SEM) of the errors collapsed across targets.

Between-subject averages (±SEM) of the collapsed signed and unsigned percentage error were shown in Fig. 5. The average collapsed signed percentage error (Fig. 5a) was consistent with an overall pattern of underestimation for all phases except for EG-Posttest’s data. For this case, the average signed percentage error was 3.4%, while for the remaining cases values were in the interval [−12.5%, −9.8%]. Differences across phases were analysed by means of *t*-tests, resulting in significant differences between Pretest and Posttest for EG’s responses [*t*(9) = 4.80, *p* = 0.0010], and between EG and CG for Posttest’s responses [*t*(18) = 3.63, *p* = 0.0019]. No significant differences were found for the remaining comparisons [CG, Pretest vs Posttest: *t*(9) = 1.74, *p* = 0.12; Pretest, CG vs EG: *t*(18) = 0.23, *p* = 0.82].

The unsigned percentage error (Fig. 5b) was consistent with a decrease of the error in EG-Posttest’s data compared to the others phases. For the former, the average unsigned percentage error was 11.1%, while for the other phases values were in the interval [15.4%, 16.4%]. However, the positive value for EG-Posttest’s data consistently indicates that EG’s participants during the Posttest phase improved their performance only partially, as they increased response accuracy for farther targets at the expense of decreasing it for nearer ones. Statistical comparisons indicated significant differences between the groups for the Posttest’s data [*t*(18) = 2.13, *p* = 0.047]. No significant differences were found for the remaining comparisons [EG, Pretest vs Posttest: *t*(9) = 1.83, *p* = 0.10; CG, Pretest vs Posttest: *t*(9) = 0.69, *p* = 0.51; Pretest, CG vs EG: *t*(18) = 0.088, *p* = 0.93].

#### A simple model for bias correction

**Figure 6:**
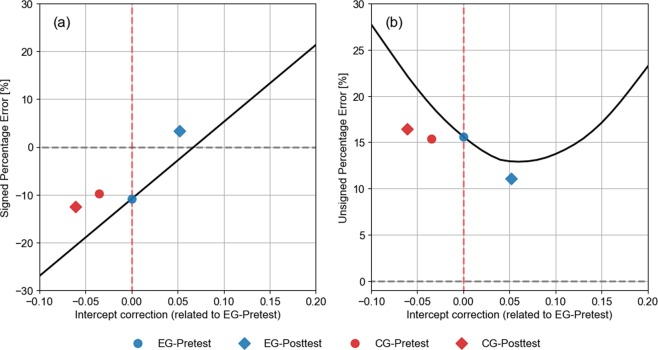
A model (black solid line) for overall reduction of the response bias. (**a**) The signed percentage error and (**b**) the unsigned percentage error values obtained by adding a constant to responses of EG-Pretest, as a function of such constant. Grey dashed lines represent the minimum error possible (0%) and red dashed line represent no intercept correction (corresponding with EG-Pretest data).

Interestingly, exploration feedback did not induce improvement of ADP precision for each test distance, but an overall reduction of the response bias. As stated before, exploration feedback induced a shift in both the signed and unsigned percentage error towards zero. Taking into account that after feedback the EG’s participants shifted the intercept while maintaining the slope relating actual and perceived distance, we investigated the effect upon the performance of applying an overall shift (change in the intercept) to the Pretest phase responses. In this way, it is possible to assess the degree of optimality of the observed feedback correction, by calculating the signed and unsigned percentage error for different values of the correction. The results are displayed in Fig. 6.

The biases (the signed and unsigned percentage error) corresponding to both groups and phases are plotted as a function of the difference between the corresponding average intercept and the intercept of the EG-Pretest’s data (note that for abscissa = 0 the markers corresponding to EG-Pretest are perfectly coincident with the curves). As it can be observed, the change of both the signed and unsigned percentage error of EG corresponds very closely to the best obtainable performance under the constraint of shifting the response to each target by the same amount: the signed percentage error approaches zero, while the unsigned percentage error approaches the minimum of the curve. Interestingly, the correction is such that the distribution of the responses midpoint (and the corresponding linear fit) is fairly coincident with the midpoint of the target range (see Fig. 3, EG-Posttest data), as one would expect theoretically (by applying, for instance, some error minimization procedure such as ordinary least-squares). In other words, subjects aimed (and were able) to minimise the error around the centre of the target range. From this point, further performance improvements can only come from changes in the slope of the response.

### Summary of main findings

In summary, the results showed that participants tend to overestimate their perceived APS boundary and underestimate the distance of sound sources located within reach. The feedback significantly reduced the response bias in both tasks. In the CG, the repetition of the task did not affect APS boundary and ADP accuracy, but it improved the performance consistency: the reachable uncertainty zone in APS was reduced and there was a tendency to decrease variability in ADP.

Taken together, the results of the APS boundary and ADP were congruent. On the one hand, in the Pretest the types of errors and their magnitude in both tasks were similar. The overestimation of the APS boundary was comparable with the perceived underestimation of the distance of the sound source. This implies that, for example, a sound source that exceeds MRD by approximately 15% would be perceived as reachable and also 15% closer than its actual position. On the other hand, the effect of feedback was also comparable in both tasks: the participants reduced the bias of their performance in the order of 10%.

## Discussion

Reaching to a sound object in the dark is an action-based response that have been scarcely studied in spatial hearing research^39–42^. Unlike the previous studies, we used reaching to measure both the APS boundary and the ADP from sources located within it, under similar conditions in a single experiment. We also tested the effect of reaching in order to touch the sound source in both tasks.

As expected, analysis of within-beyond reaching attempts showed that participants usually reach for near sound source positions and almost never reach for far targets, similarly to infant’s studies^39,40^. However, the proposed logistic model evidenced a progressive transition of responses. These results are in accordance with the existence of a gradual transition zone from near to far space, with no abrupt change at arm’s length^34,43^.

The logistic model also showed that the APS boundary was overestimated: participant responded above chance level to sound sources positioned up to 17% beyond their maximum reach. Overestimation, in this case, implies that the participant move his/her arm towards the target when, in fact, it was at a greater distance than his/her maximum reachable distance. This finding is consistent with those reported by Rosenblum *et al*.^18^. They found that the unsigned mean error on verbal estimates of auditory reachability was about 16% when participants had to imagine that they could lean forward to reach the target. It is important to note that this comparison should be done with caution, because both studies have several methodological differences –in addition to the response method–. Others studies also found that perceived visual reachability judgements were overestimated. In these cases, overestimation values were about 10% when verbal report was used^12–15,44,45^ or about 7% when a reaching response was used^17^. The difference of these findings with our results is probably due to the fact that, in spatial perception tasks, visual estimations are usually more accurate than auditory ones^46–48^. Although in some visual studies postural stability has been indicated as a factor that could affect performance, this hypothesis has been questioned^15^. Carello *et al*.^12^ evaluated the possible impact of leaning forward on a visual reaching task and found that subjects were in fact more accurate at two degrees of freedom (extending arm + bending from the hip) than at only one (extending the arm only). In addition, Rosenblum *et al*.^18^ did not find any effect of the degrees of freedom movement on auditory reachability performance.

With respect to the effect of the exploration on performance, we found that only the group that receive feedback significantly reduced incorrect responses for beyond reach positions. The data obtained with the logistic model evidenced that EG changed their perceived APS boundary after the feedback session, while it remained unchanged for the CG. The new limit was located at 6% beyond their maximum reach. Thus, data showed that the action of reaching and touching the sound source reduced the APS boundary overestimation bias in about 9.4%, presenting similar values to those obtained in vision studies that measure an action-based response without feedback^17^. Our results fit with proposals that argue that peripersonal space is structured by action^43,49^ and that the remapping of peripersonal space requires the person to have an active role in the execution of movements^10^.

Moreover, the analysis of the reachable uncertainty zone showed that the width of this zone was reduced for both groups in the Posttest phase. These data suggest that the repetition of the task induced a more consistent performance on participants, regardless of feedback. Weast and Proffit^17^ also found a similar effect and proposed that proprioceptive information could provide enough repetitive experience to improve reaching performance.

The results of the ADP task showed that, responses in the Pretest phase of both groups were almost linear with relatively high values of the regression slope (~0.8), reflecting that participants were able to perceive changes in the distance of the sound source over the entire range of tested distances. In this phase, the distance to the sound source was slightly overestimated for distances closer than ~0.4 MRD (equivalent to ~45 cm in average), while it was underestimated for greater distances. Although a similar behaviour (overestimation of near sources and underestimation of far sources) has been reported in several previous studies, in our study this transition was observed at shorter distances (~45 cm) than the ones previously reported (~1.5 m)^19–24,42,50,51^. The general methods that we used (setup, stimulus, environment, response method, etc.) differ from other studies, making difficult an exhaustive comparison. For example, in Brungart *et al*.^50^, Kopčo and Shinn-Cunningham^51^ and Parseihian *et al*.^42^ the distance attenuation intensity cue was suppressed, either by roving or by fixing the intensity at the listener’s ears in order to eliminate it during the experiments. Intensity is a dominant ADP cue, so removing it makes the task more difficult compared to our study, where the intensity was allowed to vary naturally. Another important difference is that many of the previous studies used virtual sources while here real sound sources were used. The mentioned experiments showed different ADP responses for real compared to virtual sources under similar conditions. Parseihian *et al*.^42^ studied source position pointing accuracy in the peripersonal space for both real and virtual auditory sources. Their results showed deficiency in the virtual condition compared to the real source condition. In the same direction, Brungart and Scott^24^ showed less accurate ADP responses obtained with anechoic speech signals processed with individualized head-related transfer functions, compared to those reported in earlier experiments using live talkers as stimuli^52–54^. Kerber *et al*.^20^ showed more accurate near-field ADP responses for real sources than those obtained with wave-field synthesis (virtual sources). Finally, Anderson and Zahorik^19^, Zahorik and Wightman^22^, and Zahorik^21^ conducted experiments with frontal virtual sources obtained at different distances using binaural room impulse responses. Their results showed more compressive responses (with exponent values of the power-law equal to 0.62, 0.45 and 0.39, respectively) than those obtained here (exponent = 0.85, 95% CI = [0.76, 0.94], calculated for comparison purposes).

To our knowledge, only Brungart *et al*.^23^ measured ADP in the peripersonal space using real sound sources located at near distances and by response methods similar to the one used here. The ADP curves obtained by this study were similar to those obtained in the Pretest phase, as reflected by the value of exponent obtained from a power-law fit of the responses (~0.81 vs. 0.85 respectively). However, unlike our study, in Brungart *et al*. the distance of the source was slightly overestimated for most distances. Methodological differences could explain this discrepancy. Firstly, Brungart *et al*. showed ADP curves obtained with the source located at several azimuth and elevation angles, while here the source was presented in front of the listener and at low elevations. Previous studies showed that the near-field distance accuracy depends on both azimuthal^50,51^ and elevation^50^ angles of incidence: errors are greater near the median plane and at high elevations than at lateral locations and at middle and low elevations. Secondly, in Brungart *et al*. the participants had previous visual knowledge of both the room and the experimental setup, while in our study they did not. Several authors showed that visual information can improve ADP accuracy^22,38,55^. A possible explanation to the underestimation reported here may be related to the lack of visual information about the scene, information that may have induced a slight overestimation in Brungart *et al*. In that study, the participants used an extension of their hand to mark the responses (stick pointing method) while here we used reaching. It might be possible that the response method has also contributed to the differences found between both studies. Furthermore, Brungart *et al*.’s experiment was carried out in an anechoic chamber, while here an acoustically treated room was employed.

Our experimental design aimed to achieve ecological validity regarding the stimulus (by presenting a real source fixed in amplitude located on a table), the environment (by using an acoustically treated room) and the response method (by using reaching measures). Given these aspects, combined with the fact that the ADP responses obtained in the Pretest phase are quite linear with a slope close to 1, we consider that the response that we have obtained provides a reliable baseline to measure the effect of feedback on the perception of both sound source distance and auditory peripersonal space boundary.

In the Posttest phase, the EG responses showed an approximately constant upward shift over the entire range of distances, i.e., a compensation of the bias without changes in the response range. On the contrary, the CG did not show a significant change in response bias across phases. This effect is probably due to a recalibration of the auditory field produced by the feedback. The study of the audio-tactile interaction has revealed the existence of a spatial modulation between both modalities (see review in^32^). In studies about audio-tactile ventriloquism after effects, results showed that auditory space could be rapidly recalibrated to compensate for audio-tactile spatial disparities^56^. In addition, similar results were found in a recent study that tested the effect of training with visual feedback on the bias and variability of responses to an auditory localization task in the horizontal plane^57^.

The proposed modelling of the response correction suggests that subjects who received feedback were able to apply the optimal correction under the assumption of not altering the response range (i.e. the slope). In our experiment, exploration feedback improved the global ADP accuracy (the bias) without improving the precision of the responses, as shown by the absence of a feedback effect on the intra-subject variability. The linear fit of the EG-Posttest crosses the zero bias zone very close to the middle of the range of distances used (around 0.7, range of 0.32 to 1 MRD). This indicates that participants from EG were more accurate in the middle point region of the response range in which they contacted the sound source. From this middle point, as shown in the linear model in Fig. 3c, participants seem to have calibrated their perception of the remaining distances in the Posttest phase. Although the design we used does not allow us to fully explain this effect, these results might evidence the use of an egocentrically scaled reference constructed through the feedback experience. The advantage of this calibration is that it is the simplest possible, as it allows a noticeable improvement in ADP accuracy at the cost of modifying one single response parameter. The negative aspect is that the bias decreases globally but increases locally (nearest targets are more overestimated in Posttest than Pretest). However, it is interesting to note that the slope of EG-Posttest’s linear fits showed a non-significant trend towards 1, compared to the Pretest. Although we cannot determine why participants made this correction instead of also changing the slope of the response, we might speculate that a more prolonged feedback session or a simplification of the task, could modify this pattern.

Previous studies have shown an increase in performance by auditory cues with external space without feedback, only on the basis of experience^58,59^, as subjects gained familiarity with the stimuli through trials and phases. Here the repetition of the task did not induce changes in the bias of the responses but in its intra-subject variability. The CG (the only group that performed the same task in all three phases) showed a small but significant reduction of variability across phases, while EG did not.

 The performance of participants in APS and ADP tasks was congruent. First, in the Pretest the bias observed in both tasks were similar. The linear fit of the distance perception responses crosses the perceived MRD zone at a similar distance to the perceived MRD obtained by the logistic model, showing that the response method and the experimental design proposed generated robust responses. Participants tended to overestimate in about 17% their APS boundary, and the ADP estimations from sources close to the maximum reachable distance were underestimated in a similar degree, around 15–20%. Second, the effect of the feedback is comparable. Participants presented improvement rates of their performance in the order of 10% on both tasks. Last, the repetition of the task in the CG showed similar effects: reduced the response variability of the participants.

As mentioned, both tasks have traditionally been studied separately. Weast and Proffitt^17^ proposed that the distinct bias generally found in visual reachability judgements and distance action-based estimations are possible explained by distinct underlying mechanisms that modulate actions and judgments in each task. Otherwise, it has been also suggested by Grade, Pesenti and Edwards^60^ that reachability and distance perception are both egocentric perspective tasks that involve action simulation processes. They suggest that reaching would serve as a shared metrics for egocentric space perception in reachability and distance estimates.

Our results support this last perspective and suggest that both task share egocentric frames of reference that operate in the reachable auditory peripersonal space. The complementarity of the data obtained in both tasks (proportions of biases, rates of variability and proportions of improvements after feedback) indicate, as we hypothesise that APS boundary and ADP are closely linked. It is important to note that through this dual-task, both constructs were able to be imbricated. Also, the use of normalized measures of APS boundary and ADP allowed us to taking into account the current body capacity of each participant to interact with the spatial properties of the sound source. Although, it has been shown that peripersonal space boundary is scaled to body dimensions^18,34^, the study of such scaling on ADP has not been previously addressed.

On the other hand, the effectiveness of the feedback provided is probably due to the fact that participants, when actively touch the sound object, could adjust the implied sensorimotor loop of the reaching behaviour. That is, they learnt to modulate the motor component of the task based on sensory input^61^. Participants, being able to actively check and correct their biases, were able to calibrate the motor response according to the proprioceptive and acoustic cues. This is related to recent evidence that indicates the importance of exploratory movements for the calibration of spatial hearing skills^30^.

In conclusion, our results are in line with embodied approaches of perception, that consider the role of the motor and perceptual systems, and the bodily interactions with the environment to explain cognitive phenomena (eg.^61–63^). In particular, to address perception, these approaches integrate variables related to body features and capabilities with the specific goal of the perceptual task to be performed (eg.^61,64^).

We should acknowledge some limitations of the present study. First, we used a single sound stimuli that was tested only for distances in the frontal field which might certainly limit the generalizability of our results. Second, we used only one type of feedback which make it difficult to determine whether it was specifically active exploration tactile feedback or rather the mere presence of feedback per se that induced the observed effects. In order to clarify this point, future studies should compare the effect of different types of feedback. For example, the use of passive tactile feedback (such as a finger vibrotactile stimulation) or verbal feedback on APS and ADP estimates. Also, it would be interesting to analyse and to model parameters related to changes in arm trajectory under different tasks constraints.

## Supplementary information

Supplementary Information

## Data Availability

The datasets generated during the current study are available from the corresponding author on reasonable request.
