# Supplementary material for: Reaching measures and feedback effects in auditory peripersonal space
Source: Sci Rep. 2019 Jul 1;9:9476. doi: 10.1038/s41598-019-45755-2 (PMC6603038; doi:10.1038/s41598-019-45755-2)
Supplement: Supplementary file 1 — Supplementary Information [file 41598_2019_45755_MOESM1_ESM.pdf]

Supplementary Information

Reaching measures and feedback effects in auditory peripersonal space

Mercedes X. Hüg[1,2,3,†,*], Ramiro O. Vergara[3,4,†], Fabián C. Tommasini[1,3,†], Pablo E. Etchemendy[3,4], Fernando Bermejo[1,2] & Laura G. Fernandez[1,**]

[1] Centro de Investigación y Transferencia en Acústica (CINTRA), Universidad Tecnológica Nacional - Facultad Regional Córdoba, CONICET, 5000, Córdoba, Argentina.

[2] Facultad de Psicología, Universidad Nacional de Córdoba, Argentina, 5000, Córdoba, Argentina.

[3] Consejo Nacional de Investigaciones Científicas y Tecnológicas (CONICET), Buenos Aires, Argentina.

[4] Laboratorio de Acústica y Percepción Sonora (LAPSo), Escuela Universitaria de Artes, Universidad Nacional de Quilmes, 1876, Bernal, Buenos Aires, Argentina.

[†] These authors have contributed equally to this work.

* Corresponding author: Mercedes X. Hüg (e-mail: mercehug@unc.edu.ar)

** Present address: Service de psychologie, Centre d'évaluation et de traitement de la douleur,

Hôpital Saint-Antoine, 184, rue du Faubourg Saint-Antoine, 75012, Paris, France.

Supplementary Table S1. Details for each target: distance from participant's centre, distance from participant's head, elevation from participant's head and sound level at participant's head.

Target Number	Distance from participant's centre [cm]	Distance from participant's head [cm]	Elevation from participant's head [degrees]	Sound level at participant's head [dBA SPL]
1	40	56.57	-45.0	68.4
2	45	60.21	-41.6	68.3
3	50	64.03	-38.7	68.3
4	55	68.01	-36.0	67.9
5	60	72.11	-33.7	67.4
6	65	76.32	-31.6	67.2
7	70	80.62	-29.7	66.8
8	75	85.00	-28.1	66.5
9	80	89.44	-26.6	66.2
10	85	93.94	-25.2	65.9
11	90	98.49	-24.0	65.6
12	95	103.08	-22.8	65.3
13	100	107.70	-21.8	65.0
14	105	112.36	-20.9	64.8
15	110	117.05	-20.0	64.6
16	115	121.76	-19.2	64.3
17	120	126.49	-18.4	64.1
18	125	131.24	-17.7	63.8
19	130	136.01	-17.1	63.6
20	135	140.80	-16.5	63.3
21	140	145.60	-15.9	63.1
22	145	150.42	-15.4	62.9
23	150	155.24	-14.9	62.5

Supplementary Table S2. Normalized distance intervals (bins) with its centre value, the number of trials and the number of participants averaged per each bin.

Bin centre value	Trials averaged	Number of participants averaged
0.32	44	11
0.36	80	20
0.40	80	20
0.44	80	20
0.48	80	20
0.52	24	6
0.56	80	20
0.60	80	20
0.64	80	20
0.68	80	20
0.72	80	20
0.76	60	15
0.80	80	20
0.84	64	16
0.88	80	20
0.92	80	20
0.96	80	20
1.00	80	20
1.04	80	20
1.08	80	20
1.12	80	20
1.16	64	16
1.20	80	20
1.24	56	14
1.28	36	9
1.32	36	9
1.36	16	4

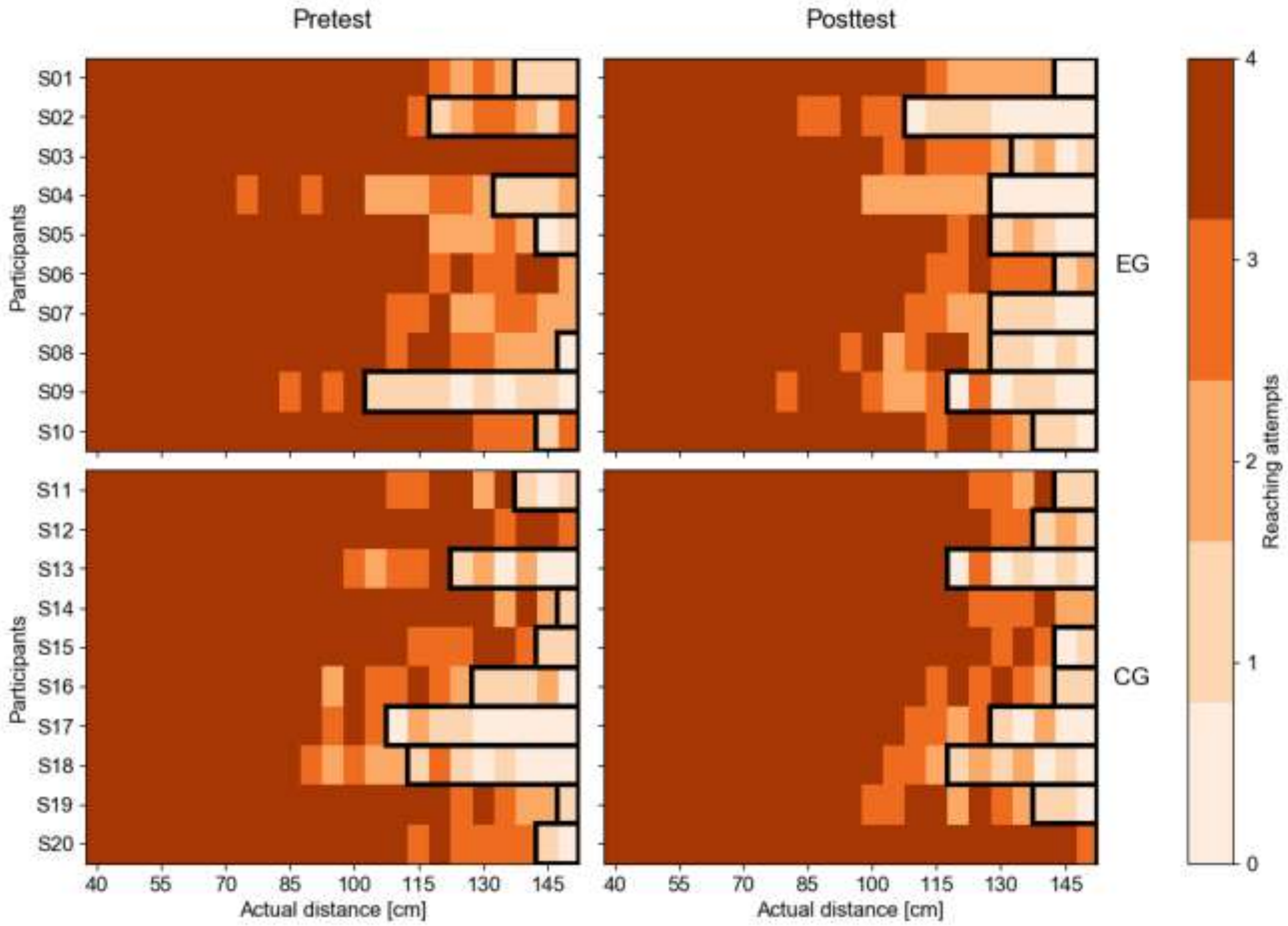

Supplementary Figure S1. Reaching attempts map for each participant by Group and Phase. Trials for each target distance (measured in cm) in which participants did not elicit a valid ADP response according to the criteria explained are indicated with black rectangles. These target distances were discarded for distance estimation analysis.

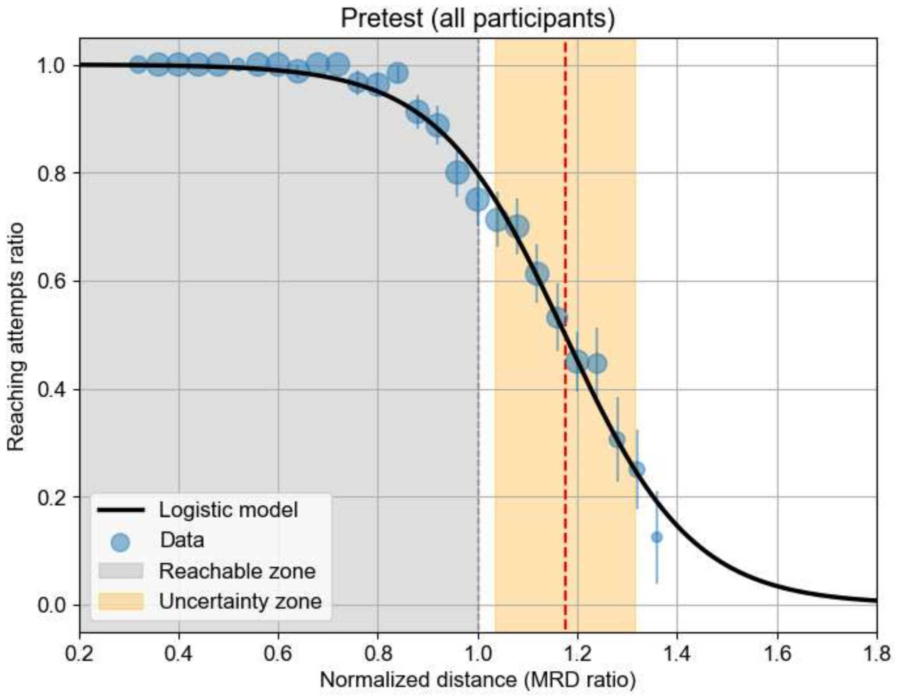

Supplementary Figure S2. Logistic model (dark black solid line) fitted to the average of reaching attempts ratio of normalized reaching distance (MRD ratio) for Pretest phase at all participants (*N* = 20). Data are presented as bubble scatter plots with SEM errors bars. Grey dashed line shows the MRD (equal to 1 in normalized distance units) and the grey area is the reachable zone. Orange zone depicts the uncertainty zone and red dashed line the perceived MRD (reaching distance at the curve inflection point).

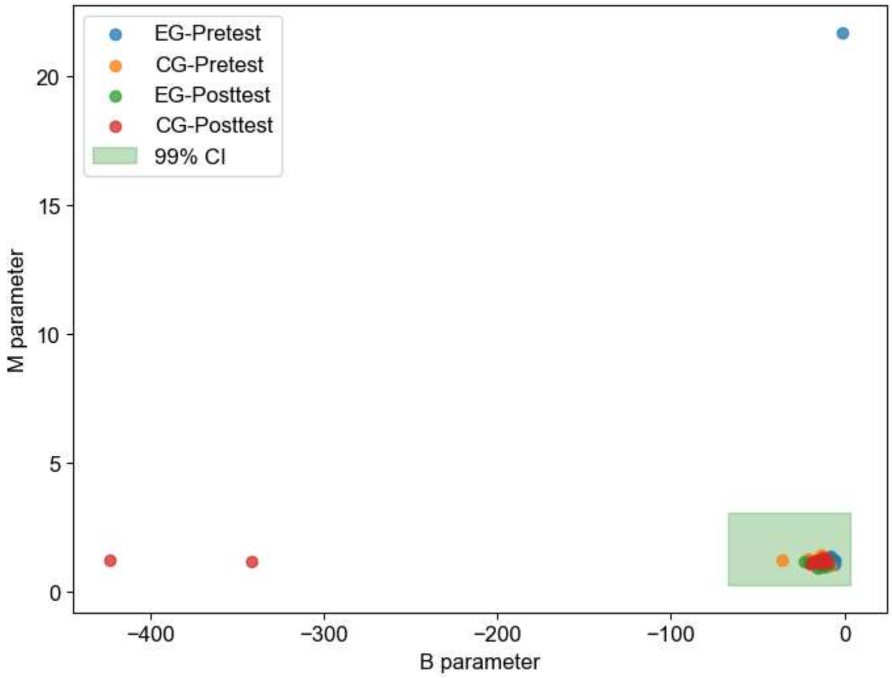

Supplementary Figure S3. Outliers analysis implemented for *B* and *M* parameter of logistic function with 99% confidence interval showed as green rectangle.

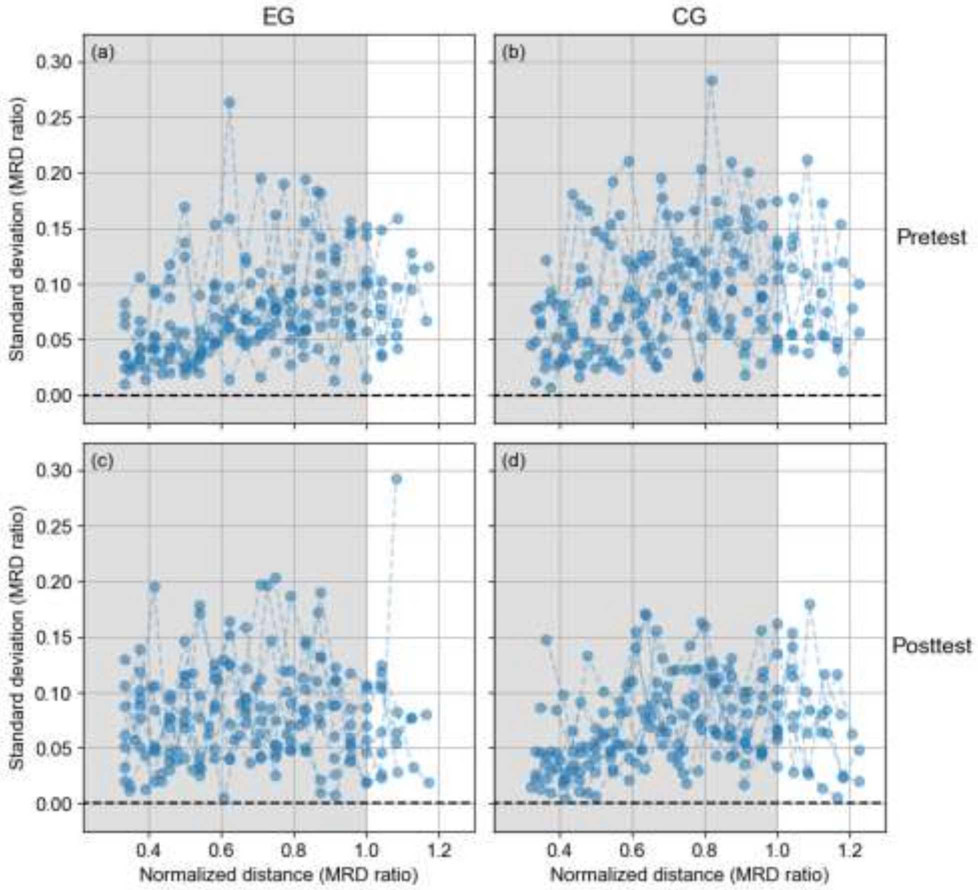

Supplementary Figure S4. Individual standard deviation (in MRD ratio) as a function of normalized source distance for (a) EG-Pretest, (b) CG-Pretest, (c) EG-Posttest, and (d) CG-Posttest. Grey area is the reachable zone.

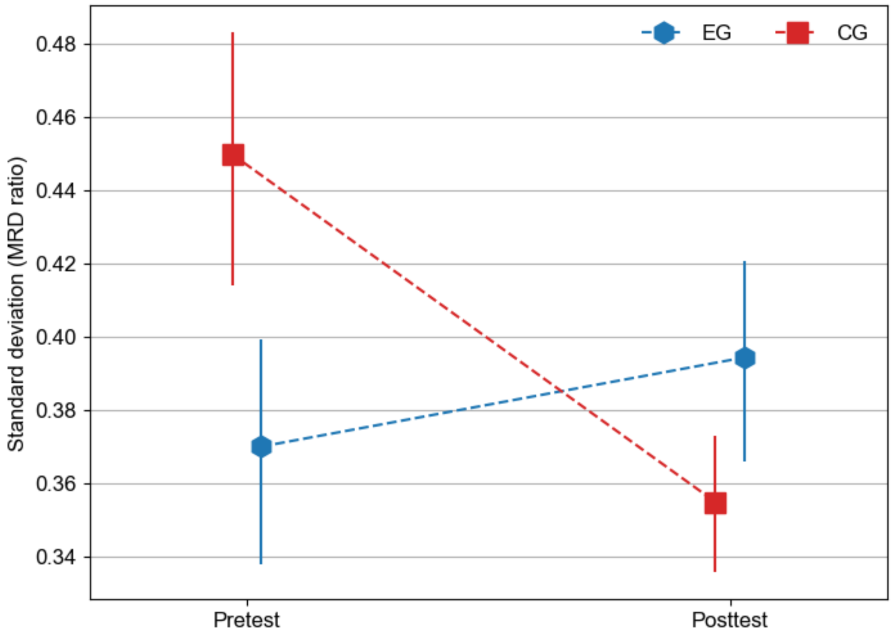

Supplementary Figure S5. Collapsed standard deviation (MRD ratio ± SEM) for both Groups across Phases for auditory distance estimation.
